# Rural factors and survival from cancer: analysis of Scottish cancer registrations

**DOI:** 10.1054/bjoc.1999.1079

**Published:** 2000-06

**Authors:** N C Campbell, A M Elliott, L Sharp, L D Ritchie, J Cassidy, J Little

**Affiliations:** Department of General Practice and Primary Care, Foresterhill Health Centre, Westburn Road, Aberdeen, AB25 2AY, UK; Department of Medicine and Therapeutics, Aberdeen University Medical School, Foresterhill, Aberdeen, AB25 2ZD, UK

**Keywords:** survival, rural, urban, cancer registry

## Abstract

In this survival study 63 976 patients diagnosed with one of six common cancers in Scotland were followed up. Increasing distance from a cancer centre was associated with less chance of diagnosis before death for stomach, breast and colorectal cancers and poorer survival after diagnosis for prostate and lung cancers. © 2000 Cancer Research Campaign


*British Journal of Cancer* (2000) **82**(11), 1863–1866
© 2000 Cancer Research Campaign
DOI: 10.1054/ bjoc.1999.1079, available online at http://www.idealibrary.com on IDEAL®

Short Communication

### Rural factors and survival from cancer: analysis of Scottish cancer registrations

**NC Campbell[1], AM Elliott[1], L Sharp[2], LD Ritchie[1], J Cassidy[2] and J Little[2]**

[1]Department of General Practice and Primary Care, Foresterhill Health Centre, Westburn Road, Aberdeen AB25 2AY, UK; [2]Department of Medicine and Therapeutics, Aberdeen University Medical School, Foresterhill, Aberdeen AB25 2ZD, UK

**Summary** In this survival study 63 976 patients diagnosed with one of six common cancers in Scotland were followed up. Increasing distance from a cancer centre was associated with less chance of diagnosis before death for stomach, breast and colorectal cancers and poorer survival after diagnosis for prostate and lung cancers. © 2000 Cancer Research Campaign

**Keywords:** survival; rural; urban; cancer registry

More than 20% of the UK population live in rural areas (Cox, 1995) but there is little information on rural–urban patterns of cancer survival (Watt et al, 1993). Studies in other countries suggest that rural residence is associated with poorer survival, which could reflect more advanced stage at diagnosis and less adjuvant treatment (Bonett et al, 1990; Liff et al, 1991; Launoy et al, 1992). In the UK, the few studies of rural health in general have produced conflicting results but, overall, challenge the widespread belief that rural people have a health advantage over their urban counterparts (Phillimore and Reading, 1992; Watt et al, 1993; Cox, 1998).

This study set out to investigate whether survival from cancer differed for patients resident in rural and urban areas. Two main rural indicators are associated with health: size of the local population and distance from health services (Weinert and Boik, 1995). In this paper, the hypotheses to be tested were that: (1) settlement size and (2) distance to the nearest cancer centre were associated with poorer survival.

**Table 1** Characteristics of cases included in analysis

| | Lung | Colorectal | Breast | Stomach | Prostate | Ovary |
|---|---|---|---|---|---|---|
| First analysis | | | | | | |
| Cases with a first primary tumour | 21 318 | 14 263 | 14 265 | 4765 | 6833 | 2532 |
| No. (%) male | 13 344 (63) | 7087 (50) | 0 (0) | 2833 (59) | 6833 (100) | 0 (0) |
| Mean (s.d.) age | 69 (10) | 70 (12) | 62 (15) | 71 (11) | 74 (9) | 64 (14) |
| No. (%) who died on date of diagnosis | 1862 (9) | 614 (4) | 445 (3) | 300 (6) | 275 (4) | 83 (3) |
| No. (%) male | 1127 (61) | 241 (39) | 0 (0) | 142 (47) | 275 (100) | 0 (0) |
| Mean (s.d.) age | 74 (10) | 78 (10) | 82 (11) | 76 (10) | 80 (8) | 75 (12) |
| Second analysis | | | | | | |
| Cases (first tumour) followed up for at least 1 day[a] | 19 449 | 13 645 | 13 817 | 4464 | 6555 | 2449 |
| No. (%) male | 12 214 (63) | 6844 (50) | 0 (0) | 2690 (60) | 6555 (100) | 0 (0) |
| Mean (s.d.) age | 69 (10) | 70 (11) | 61 (14) | 70 (11) | 73 (9) | 64 (14) |
| No. (%) who died on or before 31 December 1995 | 16 433 (84) | 6495 (48) | 2940 (21) | 3479 (78) | 2644 (40) | 1406 (57) |
| No. (%) male | 10 343 (63) | 3252 (50) | 0 (0) | 2093 (60) | 2644 (100) | 0 (0) |
| Mean (s.d.) age | 69 (10) | 72 (12) | 68 (15) | 71 (11) | 76 (9) | 68 (12) |

[a]Excludes 3579 cases who died on the first day and 18 other cases who were followed up for less than 1 day.

*Received 9 September 1999*
*Revised 26 November 1999*
*Accepted 26 November 1999*

*Correspondence to:* NC Campbell

**Table 2** Numbers and percentages of patients who were diagnosed on their date of death

| | Lung | | Colorectal | | Breast | | Stomach | | Prostate | | Ovary | |
|---|---|---|---|---|---|---|---|---|---|---|---|---|
| | *n* | % | *n* | % | *n* | % | *n* | % | *n* | % | *n* | % |
| Carstairs deprivation quintile | | | | | | | | | | | | |
| 1 – least deprived | 190/2224 | 8.5 | 125/2635 | 4.7 | 102/3057 | 3.3 | 37/623 | 5.9 | 47/1384 | 3.4 | 16/500 | 3.2 |
| 2 | 290/3363 | 8.6 | 126/3049 | 4.1 | 103/3008 | 3.4 | 60/870 | 6.9 | 70/1558 | 4.5 | 15/529 | 2.8 |
| 3 | 389/4564 | 8.5 | 120/3047 | 3.9 | 87/3037 | 2.9 | 54/1059 | 5.1 | 60/1488 | 4.0 | 18/575 | 3.1 |
| 4 | 500/5710 | 8.8 | 135/3101 | 4.4 | 98/2953 | 3.3 | 71/1183 | 6.0 | 61/1433 | 4.3 | 17/535 | 3.2 |
| 5 – most deprived | 492/5445 | 9.0 | 108/2427 | 4.4 | 55/2199 | 2.5 | 78/1026 | 7.6 | 37/968 | 3.8 | 17/393 | 4.3 |
| *P*-value[a] | 0.388 | | 0.777 | | 0.124 | | 0.312 | | 0.662 | | 0.371 | |
| Distance to cancer centre | | | | | | | | | | | | |
| ≤ 5 km | 435/5526 | 7.9 | 122/3313 | 3.7 | 74/3023 | 2.4 | 51/1135 | 4.5 | 55/1536 | 3.6 | 14/585 | 2.4 |
| 6–13 km | 415/4520 | 9.2 | 107/2711 | 3.9 | 66/2696 | 2.4 | 64/987 | 6.5 | 51/1223 | 4.2 | 14/493 | 2.8 |
| 14–23 km | 386/3764 | 10.3 | 126/2557 | 4.9 | 80/2738 | 2.9 | 65/875 | 7.4 | 54/1202 | 4.5 | 20/486 | 4.1 |
| 24–37 km | 321/3786 | 8.5 | 119/2568 | 4.6 | 100/2854 | 3.5 | 52/855 | 6.1 | 47/1371 | 3.4 | 15/478 | 3.1 |
| > 38 km | 305/3722 | 8.2 | 140/3114 | 4.5 | 125/2954 | 4.2 | 68/913 | 7.4 | 68/1501 | 4.5 | 20/490 | 4.1 |
| *P*-value[a] | 0.671 | | 0.046 | | <0.001 | | 0.016 | | 0.429 | | 0.127 | |
| Settlement size | | | | | | | | | | | | |
| > 1 000 000 | 670/7042 | 9.5 | 158/3567 | 4.4 | 119/3562 | 3.3 | 113/1390 | 8.1 | 56/1443 | 3.9 | 21/649 | 3.2 |
| 100 000–1 000 000 | 265/3563 | 7.4 | 100/2569 | 3.9 | 49/2393 | 2.0 | 36/847 | 4.3 | 53/1347 | 3.9 | 12/464 | 2.6 |
| 10 000–100 000 | 498/5783 | 8.6 | 169/3879 | 4.4 | 130/4048 | 3.2 | 76/1300 | 5.8 | 68/1860 | 3.7 | 22/738 | 3.0 |
| 1000–10 000 | 292/3332 | 8.8 | 116/2661 | 4.4 | 82/2687 | 3.1 | 42/827 | 5.1 | 59/1279 | 4.6 | 17/429 | 4.0 |
| < 1000 | 137/1598 | 8.6 | 71/1587 | 4.5 | 65/1575 | 4.1 | 33/401 | 8.2 | 39/904 | 4.3 | 11/252 | 4.4 |
| *P*-value[a] | 0.203 | | 0.790 | | 0.145 | | 0.194 | | 0.396 | | 0.293 | |

[a]Chi-square test for linear trend.

**Table 3** Odds ratios (OR) (95% confidence intervals (CI)) for death on date of diagnosis, adjusted for age, sex, distance to cancer centre and settlement size

| | Lung | | Colorectal | | Breast | | Stomach | | Prostate | | Ovary | |
|---|---|---|---|---|---|---|---|---|---|---|---|---|
| | OR | 95% CI | OR | 95% CI | OR | 95% CI | OR | 95% CI | OR | 95% CI | OR | 95% CI |
| Distance to cancer centre | | | | | | | | | | | | |
| ≤ 5 km | 1 | – | 1 | – | 1 | – | 1 | – | 1 | – | 1 | – |
| 6–13 km | 1.17 | (1.01–1.36) | 1.18 | (0.89–1.56) | 1.20 | (0.83–1.73) | 1.52 | (1.03–2.26) | 1.39 | (0.92–2.10) | 1.30 | (0.58–2.93) |
| 14–23 km | 1.47 | (1.22–1.77) | 1.92 | (1.35–2.71) | 2.15 | (1.41–3.30) | 2.83 | (1.77–4.53) | 1.46 | (0.85–2.51) | 2.69 | (1.01–7.14) |
| 24–37 km | 1.21 | (0.97–1.52) | 1.86 | (1.25–2.76) | 2.65 | (1.62–4.34) | 3.15 | (1.78–5.57) | 1.10 | (0.60–2.02) | 1.95 | (0.63–6.01) |
| ≥ 38 km | 1.14 | (0.90–1.43) | 1.78 | (1.19–2.67) | 2.87 | (1.74–4.74) | 3.92 | (2.16–7.08) | 1.36 | (0.75–2.47) | 2.47 | (0.79–7.65) |
| *P*-value: | | | | | | | | | | | | |
| Global | | < 0.001 | | 0.006 | | < 0.001 | | < 0.001 | | 0.347 | | 0.325 |
| Linear trend | | 0.512 | | 0.024 | | < 0.001 | | < 0.001 | | 0.529 | | 0.263 |
| Settlement size | | | | | | | | | | | | |
| > 1 000 000 | 1 | – | 1 | – | 1 | – | 1 | – | 1 | – | 1 | – |
| 100 000–1000 000 | 0.78 | (0.67–0.92) | 0.90 | (0.68–1.19) | 0.54 | (0.37–0.78) | 0.64 | (0.45–1.05) | 1.14 | (0.76–1.74) | 1.02 | (0.46–2.29) |
| 10 000–100 000 | 0.81 | (0.68–0.96) | 0.66 | (0.48–0.90) | 0.51 | (0.34–0.75) | 0.36 | (0.24–0.58) | 0.94 | (0.57–1.55) | 0.63 | (0.27–1.48) |
| 1000–10 000 | 0.83 | (0.68–1.01) | 0.64 | (0.45–0.90) | 0.42 | (0.27–0.64) | 0.28 | (0.18–0.50) | 1.12 | (0.67–1.87) | 0.75 | (0.30–1.86) |
| < 1000 | 0.84 | (0.66–1.07) | 0.72 | (0.49–1.06) | 0.54 | (0.34–0.87) | 0.45 | (0.26–0.85) | 1.01 | (0.58–1.78) | 0.90 | (0.33–2.43) |
| *P*-value: | | | | | | | | | | | | |
| Global | | 0.009 | | 0.088 | | < 0.001 | | < 0.001 | | 0.867 | | 0.812 |
| Linear trend | | 0.016 | | 0.066 | | 0.001 | | < 0.001 | | 0.670 | | 0.952 |

### METHODS

Data on lung, colorectal, breast, prostate, stomach and ovarian cancers diagnosed between 1 January 1991 and 31 December 1995 were obtained from the Scottish cancer registry. Based on post-code of residence at diagnosis, 70 561 of 71 152 registrations were successfully matched to census output areas, which were used to assign geographical and socio-economic variables (output areas are the smallest census units in Scotland – median population 124, interquartile range 98–156). Cases registered with a first primary tumour (63 976) were eligible for analysis (Table 1).

Survival was calculated from date of diagnosis to date of death or 31 December 1995, whichever was sooner (median follow up 0.68 years; range 0–5 years). Distance quintiles were assigned based on the shortest straight-line distance to the nearest cancer centre. The quintiles represented ≤ 5 km, 6–13 km, 14–23 km, 24–37 km and ≥ 38 km. Census indicators of settlement size were used, representing populations of > 1 000 000, 100 000–1 000 000, 10 000–100 000, 1000–10 000 and < 1000. Deprivation scores were calculated using the method of Carstairs and Morris (1990) but with output areas as the geographical units. Quintiles were calculated with the least deprived coded ‘1’, and the most deprived coded ‘5’ (Reading et al, 1993). Deprivation indices could not be assigned for 33 cases due to missing census data.

© 2000 Cancer Research Campaign

**Table 4** One-year survival[a] of patients who survived at least 1 day from their date of diagnosis.

| | Lung | | Colorectal | | Breast | | Stomach | | Prostate | | Ovary | |
|---|---|---|---|---|---|---|---|---|---|---|---|---|
| | No. starting | 1-year survival (%) | No. starting | 1-year survival (%) | No. starting | 1-year survival (%) | No. starting | 1-year survival (%) | No. starting | 1-year survival (%) | No. starting | 1-year survival (%) |
| Carstairs deprivation quintile | | | | | | | | | | | | |
| 1 – least deprived | 2033 | 24.0 | 2509 | 68.5 | 2955 | 92.5 | 586 | 28.9 | 1336 | 80.1 | 484 | 61.3 |
| 2 | 3072 | 23.5 | 2923 | 65.8 | 2904 | 91.0 | 810 | 30.6 | 1487 | 77.1 | 514 | 55.9 |
| 3 | 4175 | 21.2 | 2927 | 65.1 | 2949 | 88.3 | 1004 | 29.3 | 1427 | 76.3 | 557 | 56.1 |
| 4 | 5207 | 20.6 | 2964 | 62.5 | 2854 | 88.3 | 1112 | 26.2 | 1372 | 75.8 | 518 | 51.7 |
| 5 – most deprived | 4951 | 21.2 | 2318 | 62.2 | 2144 | 86.1 | 948 | 28.9 | 931 | 71.9 | 376 | 50.8 |
| *P*-value[b] | < 0.001 | | < 0.001 | | < 0.001 | | 0.283 | | < 0.001 | | < 0.001 | |
| Distance to cancer centre | | | | | | | | | | | | |
| ≤ 5 km | 5090 | 21.7 | 3190 | 65.4 | 2949 | 87.8 | 1084 | 28.8 | 1480 | 74.9 | 571 | 58.3 |
| 6–13 km | 4104 | 21.6 | 2603 | 63.6 | 2629 | 90.3 | 923 | 28.6 | 1172 | 76.3 | 479 | 55.4 |
| 14–23 km | 3377 | 21.9 | 2431 | 65.0 | 2657 | 89.9 | 810 | 30.4 | 1148 | 76.8 | 466 | 52.2 |
| 24–37 km | 3461 | 21.0 | 2448 | 65.1 | 2754 | 90.2 | 802 | 26.4 | 1323 | 78.5 | 463 | 55.7 |
| ≥ 38 km | 3417 | 22.1 | 2973 | 64.9 | 2828 | 89.0 | 845 | 29.1 | 1432 | 76.5 | 470 | 54.1 |
| *P*-value[b] | 0.862 | | 0.174 | | 0.208 | | 0.438 | | 0.908 | | 0.950 | |
| Settlement size | | | | | | | | | | | | |
| > 1 000 000 | 6370 | 20.2 | 3409 | 62.4 | 3442 | 87.6 | 1277 | 27.1 | 1387 | 70.4 | 628 | 51.7 |
| 100 000–1 000 000 | 3298 | 23.1 | 2467 | 65.6 | 2344 | 89.6 | 811 | 28.8 | 1293 | 78.4 | 452 | 61.8 |
| 10 000–1000 000 | 5280 | 21.7 | 3708 | 65.9 | 3917 | 89.9 | 1224 | 29.6 | 1791 | 79.3 | 716 | 53.3 |
| 1000–10 000 | 3040 | 22.0 | 2545 | 64.9 | 2604 | 90.2 | 785 | 30.2 | 1219 | 77.4 | 412 | 54.7 |
| < 1000 | 1461 | 23.7 | 1516 | 66.1 | 1510 | 90.5 | 367 | 27.6 | 865 | 76.8 | 241 | 60.5 |
| *P*-value[b] | < 0.001 | | < 0.001 | | 0.001 | | 0.021 | | < 0.001 | | 0.071 | |

[a]Calculated using the Kaplan–Meier method. [b]Log-rank test for trend.

**Table 5** Proportional hazard ratios (95% confidence intervals) for survival after diagnosis, adjusted for age, sex, deprivation, distance to cancer centre and settlement size

| | Lung | | Colorectal | | Breast | | Stomach | | Prostate | | Ovary | |
|---|---|---|---|---|---|---|---|---|---|---|---|---|
| | HR | 95% CI | HR | 95% CI | HR | 95% CI | HR | 95% CI | HR | 95% CI | HR | 95% CI |
| Distance to cancer centre | | | | | | | | | | | | |
| ≤ 5 km | 1 | – | 1 | – | 1 | – | 1 | – | 1 | – | 1 | – |
| 6–13 km | 1.03 | (0.98–1.08) | 1.06 | (0.98–1.15) | 0.87 | (0.77–0.98) | 1.02 | (0.92–1.13) | 1.13 | (1.00–1.28) | 1.13 | (0.95–1.34) |
| 14–23 km | 1.07 | (1.01–1.14) | 1.09 | (0.99–1.21) | 0.99 | (0.86–1.15) | 1.09 | (0.95–1.26) | 1.10 | (0.94–1.30) | 1.33 | (1.06–1.65) |
| 24–37 km | 1.09 | (1.02–1.18) | 1.11 | (0.99–1.25) | 1.04 | (0.88–1.22) | 1.18 | (1.01–1.38) | 1.01 | (0.84–1.21) | 1.20 | (0.94–1.54) |
| ≥ 38 km | 1.09 | (1.01–1.18) | 1.11 | (0.99–1.24) | 1.07 | (0.90–1.27) | 1.13 | (0.96–1.33) | 1.23 | (1.02–1.48) | 1.15 | (0.89–1.49) |
| *P*-value: | | | | | | | | | | | | |
| Global | | 0.160 | | 0.355 | | 0.057 | | 0.322 | | 0.009 | | 0.118 |
| Linear trend | | 0.024 | | 0.108 | | 0.301 | | 0.122 | | 0.042 | | 0.562 |
| Settlement size | | | | | | | | | | | | |
| > 1 000 000 | 1 | – | 1 | – | 1 | – | 1 | – | 1 | – | 1 | – |
| 100 000–1 000 000 | 0.94 | (0.89–0.99) | 0.92 | (0.85–0.99) | 0.84 | (0.74–0.95) | 0.94 | (0.84–1.05) | 0.74 | (0.65–0.84) | 0.87 | (0.73–1.04) |
| 10 000–100 000 | 0.93 | (0.87–0.99) | 0.87 | (0.79–0.96) | 0.81 | (0.70–0.93) | 0.83 | (0.73–0.95) | 0.73 | (0.63–0.85) | 0.89 | (0.72–1.08) |
| 1000–10 000 | 0.91 | (0.85–0.98) | 0.87 | (0.78–0.97) | 0.86 | (0.74–1.00) | 0.78 | (0.68–0.90) | 0.79 | (0.67–0.93) | 0.80 | (0.64–1.00) |
| < 1000 | 0.86 | (0.79–0.93) | 0.90 | (0.80–1.02) | 0.86 | (0.72–1.03) | 0.92 | (0.77–1.09) | 0.80 | (0.67–0.96) | 0.82 | (0.63–1.07) |
| *P*-value: | | | | | | | | | | | | |
| Global | | 0.002 | | 0.023 | | 0.005 | | 0.005 | | < 0.001 | | 0.235 |
| Linear trend | | <0.001 | | 0.052 | | 0.044 | | 0.027 | | 0.014 | | 0.039 |

Data were managed using Microsoft Access version 2 and analysed using SPSS for Windows release 7. Proportions of cases whose date of diagnosis coincided with their date of death were calculated. Logistic regression was used to model all variables and calculate odds ratios relative to the first category within each variable. Survival rates after diagnosis were calculated by Kaplan–Meier analysis (Bland and Altman, 1998). Cox regression was used to model all variables and calculate hazard ratios relative to the first category within each variable (Cox, 1972).

### RESULTS

In univariate analysis, a greater proportion of patients who lived far from a cancer centre died on their date of diagnosis compared

*© 2000 Cancer Research Campaign*

with those who lived nearby (Table 2). Trends were significant for colorectal, breast and stomach cancers and persisted after adjusting for age, sex and settlement size (Table 3). In the modelling exercise, small settlement size was an advantage for all sites except prostate and these trends were significant for lung, breast and stomach. The effect was, however, largely evident as a difference between the first category (patients living in a conurbation of more than one million) and the rest.

For patients who survived at least 1 day after diagnosis, increasing deprivation was associated with decreasing survival for all sites except stomach (Table 4). Small settlement size was a significant advantage for all sites except ovary. Adjusting for age, sex, deprivation and distance, the survival advantage associated with small settlement size was confirmed, although the effect was again mostly seen between patients living in a conurbation of more than one million and the rest. Increasing distance from a cancer centre was significantly associated with poorer survival for lung and prostate cancers.

### DISCUSSION

Cancer registration data in Scotland have a high level of accuracy compared to other registries. In comparison with medical records, serious discrepancies were judged to have occurred in under 3% of cases and postcode inaccuracies in 7% (Brewster et al, 1994). Our findings could have been affected by bias if cancer registry data, which are collected by case notes review, were less complete for more remote patients. Several factors suggest that this is unlikely. First, common methods of case ascertainment and registration are used throughout Scotland, irrespective of where cases are resident. Second, all records of deceased patients are collected together in central stores so are equally accessible. Third, if our findings were due to bias, they should have been consistent across all cancers, which they were not. Finally, registration bias would not explain the trend to poorer survival after diagnosis.

Standard area-based indicators of deprivation have been criticised as insensitive in rural areas, where wealth and poverty can coexist in close proximity. We minimized internal diversity by using the smallest available area unit, an approach that has been found effective at showing inequalities even in rural areas (Reading et al, 1993).

### Interpretation

We found no evidence that small settlement size was a survival disadvantage. The only prominent association was poorer survival for patients living in a conurbation of more than one million. In Scotland, this is the extended Glasgow area and it seems likely that local factors, perhaps including deprivation incompletely controlled for by the Carstairs score, could have been responsible. In the rest of Scotland, settlement size had little or no effect.

There was, however, strong evidence that increasing distance from a cancer centre was associated with poorer survival. More remote patients were less likely to be diagnosed before they died, especially for stomach, breast and colorectal cancers. After diagnosis, there appeared to be a small disadvantage with increasing distance, although this association was weaker.

Studies in other countries have found that patients with poor access were more likely to present with disseminated disease for breast, colorectal, prostate and lung cancers (Liff et al, 1991; Launoy et al, 1992; Montella et al, 1995). They have also been found less likely to be referred to specialist centres (Greenberg et al, 1988*a*; Launoy et al, 1992) or receive adjuvant treatment with radiotherapy and chemotherapy (Greenberg et al 1988*b*; Craft et al, 1997). In Scotland, rural residence has been associated with suboptimal treatment for testicular cancer (Howard et al, 1995). Our findings are in line with these studies and suggest that the problem is primarily one of distance from cancer centres. If these findings are confirmed by further research, and equity of access to cancer treatment remains a priority (EAGC, 1995), then changes may be needed to ease and streamline referral and treatment for more remote patients.

### ACKNOWLEDGEMENTS

We thank everyone at the Scottish cancer registry (Information and Statistics Division of the Scottish Health Service) and in particular Veronica Harris, who extracted the data we sought on Scottish cancer registrations. Neil Campbell is funded by a Cancer Research Campaign Primary Care Oncology Fellowship.

### REFERENCES

Bland MJ and Altman DG (1998) Statistics notes. Survival probabilities (the Kaplan–Meier method). *Br Med J* **317**: 1572

Bonett A, Dorsch M, Roder D and Esterman A (1990) Infiltrating ductal carcinoma of the breast in South Australia. Implications of trends in tumour diameter, nodal status and case-survival rates for cancer control. *Med J Aust* **152**: 19–23

Brewster D, Crichton J and Muir C (1994) How accurate are Scottish cancer registration data? *Br J Cancer* **70**: 954–959

Carstairs V and Morris R (1990) *Deprivation and Health in Scotland*. University Press: Aberdeen

Cox D (1972) Regression models and life tables. *J R Stat Soc* **34**: 187–220

Cox J (1995) *Rural General Practice in the United Kingdom. Occasional Paper 71*. The Royal College of General Practitioners: London

Cox J (1998) Poverty in rural areas. *Br Med J* **316**: 722

Craft PS, Primrose JG, Lindner JA and McManus PR (1997) Surgical management of breast cancer in Australian women in 1993: analysis of Medicare statistics. *Med J Aust* **166**: 626–629

Expert Advisory Group on Cancer (1995) *A Policy Framework for Commissioning Cancer Services: a Report by the Expert Advisory Group on Cancer to the Chief Medical Officers of England and Wales, 1995*. HM Stationery Office: London

Greenberg ER, Dain B, Freeman D, Yates J and Korson R (1988*a*) Referral of lung cancer patients to university hospital cancer centers. A population-based study in two rural states. *Cancer* **62**: 1647–1652

Greenberg ER, Chute CG, Stukel T, Baron JA, Freeman DH, Yates J and Korson R (1988*b*) Social and economic factors in the choice of lung cancer treatment. A population-based study in two rural states. *N Engl J Med* **318**: 612–617

Howard GCW, Clarke K, Elia MH, Hutcheon AW, Kaye SB, Windsor PM Yosef HMA and Sharp L (1995) A Scottish national mortality study assessing cause of death, quality of and variation in management of patients with testicular non-seminomatous germ-cell tumours. *Br J Cancer* **72**: 1307–1311

Launoy G, Le Coutour X, Gignoux M, Pottier D and Dugleux G (1992) Influence of rural enviornment on diagnosis, treatment, and prognosis of colorectal cancer. *J Epidemiol Comm Health* **46**: 365–367

Liff JM, Chow WH and Greenberg RS (1991) Rural–urban differences in stage at diagnosis. Possible relationship to cancer screening. *Cancer* **67**: 1454–1459

Montella M, Biondi E, De Marco M, Botti G, Tatangelo F, Capasso I and Marone A (1995) Sociodemographic factors associated with the diagnostic staging of breast cancer in southern Italy. *Cancer* **76**: 1585–1590

Phillimore P and Reading R (1992) A rural advantage? Urban–rural health differences in Northern England. *J Pub Health Med* **14**: 290–299

Reading R, Jarvis S and Openshaw S (1993) Measurement of social inequalities in health and use of health services among children in Northumberland. *Arch Dis Child* **68**: 626–631

Watt IS, Franks AJ and Sheldon TA (1993) Rural health and health care. *Br Med J* **306**: 1358–1359

Weinert C and Boik J (1995) MSU rurality index: development and evaluation. *Research Nurs Health* **18**: 453–464

© 2000 Cancer Research Campaign

